# Lung cancer: a nationwide analysis of sex and age incidence trends from 1980 to 2022

**DOI:** 10.2340/1651-226X.2024.34876

**Published:** 2024-06-30

**Authors:** Morten Borg, Hanne Tønnesen, Rikke Ibsen, Ole Hilberg, Anders Løkke

**Affiliations:** a Department of Medicine, Lillebaelt Hospital Vejle, Beriderbakken, Denmark; b WHO-CC, the Parker Institute, Bispebjerg-Frederiksberg Hospital, Copenhagen University, Copenhagen, Denmark; c I2Minds, Aarhus, Denmark; d Department of Medicine, Lillebaelt Hospital Vejle, Vejle, Denmark

**Keywords:** Lung cancer, incidence, epidemiology, female, male, incidence forecasting

## Abstract

**Background:**

Lung cancer, once rare, has evolved into the global leading cause of cancer-related mortality, primarily driven by widespread cigarette smoking in the 20th century. This study explores the historical trends of lung cancer incidence in Denmark over four decades, emphasizing the impact of smoking prevalence, age, and gender on the observed patterns.

**Materials and methods:**

Drawing upon data from the Danish National Patient Register and information on smoking habits provided by the Danish Health Authority, this study investigates lung cancer incidence rates, demographic shifts, and smoking prevalence from 1980 to 2022.

**Results:**

Smoking prevalence exhibited a consistent decline in males from 1950 to 2022, whereas female smoking prevalence maintained a stable level from 1950 to 1987, followed by a subsequent decline from 1987 to 2022. A peak in lung cancer crude incidence rates was identified during 2014–2017, with no significant difference observed before and after this period. Over the period, the gender distribution transitioned from a male majority to an equal male-female ratio, and age-specific disparities indicated declines in patients aged 50–59 and increases in those above 80 years.

**Interpretation:**

The certainty of a decline in lung cancer incidence in the coming years remains unclear. Based on smoking prevalence, it might still be a decade away. To ensure a sustained decline in lung cancer incidence, targeted interventions are imperative, including customized smoking cessation programs that could be designed favorably for females. Given the modest decline in smoking prevalence over the last decade, legislation aimed at discouraging young individuals from smoking is pivotal. As of now, these efforts have not been implemented in Denmark.

## Introduction

Lung cancer has emerged as the leading cause of cancer-related deaths globally [1], marking a significant historical shift from its rare occurrence at the onset of the 20th century [2] to its current status as the most prevalent cancer worldwide [1]. This transformation is directly linked to the widespread adoption of cigarette smoking throughout the 20th century [3, 4], exemplified by the exponential surge in lung cancer cases. The time lapse between smoking initiation and lung cancer onset varies due to factors such as genetic predisposition, cumulative smoking exposure, and early onset of smoking during adolescence [5]. While tobacco smoking, both active and passive, remains the primary contributor to lung cancer in Western populations [6], other factors like legislation, ethnicity, genetics, and life expectancy also play roles [7].

Notably, regional disparities in lung cancer rates, with Eastern Europe exhibiting the highest incidence among males and North America, Australia, and Western Europe among females, are primarily attributed to differing levels of smoking exposure [8]. However, unique genetic susceptibilities contribute to disparities, as seen in certain populations like African Americans, Polynesians, and South Korean women [9–11]. The hypothesis of our study suggests a peak and subsequent decline in lung cancer incidence in Denmark, aligning with historical smoking trends [12]. Over the last decade, Denmark has seen approximately 5,000 cases of lung cancer annually. We aim to analyze lung cancer trends over four decades in Denmark, exploring gender and age-specific variations and identifying factors contributing to high rates among Danish women.

## Material and methods

The Danish National Health Service provides tax-funded universal health care [13]. Upon birth or immigration, every Danish resident is assigned a unique 10-digit Civil Registration System (CPR) number, which enables precise individual-level record linkage across national registries and lifelong follow-up [14].

Patients aged 18 years or older who were diagnosed with lung cancer between January 1, 1980, and December 31, 2022, were identified in the Danish National Patient Register (DNPR). The DNPR, an administrative registry, has achieved complete nationwide coverage for all non-psychiatric diagnoses since 1978 [14]. Registration, which is mandatory and submitted by treating physicians, serves the purpose of continuously monitoring hospital and health service utilization and facilitating billing.

Primary and secondary diagnoses are classified according to the International Classification of Diseases and Related Health Problems (ICD) [15]. During the period from 1980 to 1993, lung cancer cases were identified using ICD 8th revision (ICD-8) codes 163 and 164. From 1994 to 2022, ICD-10 codes c33–c34 were used. The CPR number reveals both the age and sex of individuals.

Smoking habits in Denmark were provided by the Danish Health Authority [16]. Data were collected by Gallup and PLS Consult at the request of organizations such as the Danish Heart Foundation, the Danish Cancer Society, the Council on Tobacco Damage, the Danish Health Authority, and the Danish Lung Foundation. The data collection was conducted through random sampling across cohorts of varying sizes.

Analysis of differences in incidence rate was calculated as incidence rate ratio with 95% confidence intervals using Exact Poisson Method. Analyses were performed using R statistical software (Fox & Leanage, 2016) and graphs were produced in Microsoft Excel version 16.71.

## Results

Table 1 illustrates the attributes of lung cancer patients across three distinct time spans: 1980–1993, 1994–2007, and 2008–2018. Notably, during the initial period (1980–1993), the majority of patients were males, while the sex distribution approached a near balance of 50/50 in the most recent interval (*p* < 0.01). The analysis of age distribution reveals a decline in patients aged 50–59 years between 1980–1993 and 2007–2018, along with a concurrent rise in patients aged above 80 years (*p* < 0.01).

**Table 1:** Attributes of lung cancer patients across three distinct time spans: 1980–1993, 1994–2007, and 2008–2018.

Variable	1980–1993	1994–2007	2008–2018
Lung cancer patients	53,056	56,071	53,869
Males (%)	67.4	56.0	50.8
Females (%)	32.6	44.0	49.2
Mean age at diagnosis			
All patients (years)	67.7	68.3	70.4
Males (years)	68.2	68.8	70.7
Females (years)	66.4	67.6	70.1
Age groups			
< 50 years (%)	5.7	4.9	2.6
50–59 years (%)	15.4	15.7	11.4
60–69 years (%)	32.8	30.4	30.7
70–79 years (%)	33.6	34.5	36.1
80+ years (%)	12.5	14.5	19.2

**Figure 1:**
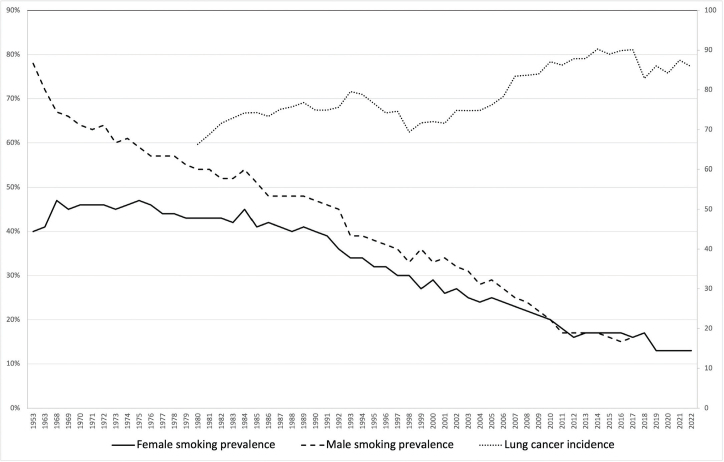
Prevalence of daily smoking (left y-axis) and incidence of lung cancer per 100,000 inhabitants (right y-axis).

Figure 1 illustrates the prevalence of daily smoking in the general population of Denmark for both males and females as well as the crude incidence of lung cancer per 100,000 inhabitants. In 1950, approximately 78% of the male population were smokers. Over the course of the following five decades, a gradual decrease in smoking prevalence was noted, resulting in a consistent smoking rate of around 17% from 2012 to 2018. Subsequently, there was a further decline to 13% in the years 2020–2022. In contrast, the prevalence of daily smoking among the female population showed an increase from the 1950s to the late 1970s, with rates ranging between approximately 40 and 45%. However, a significant decline has been observed since 1980, leading to smoking prevalence levels comparable to those of men from 2012 to 2022.

The crude lung cancer incidence rate per 100,000 inhabitants ranged from 66 to 77 per 100,000 inhabitants in the 1980s and gradually climbed to a peak of 90 per 100,000 inhabitants during 2014–2017 followed by a decline to 84–86 per 100,000 inhabitants. The incidence rate ratio before and after the peak (periods 2009–2013 and 2018–2022) was 1.06 (95% confidence interval 0.98–1.06).

**Figure 2:**
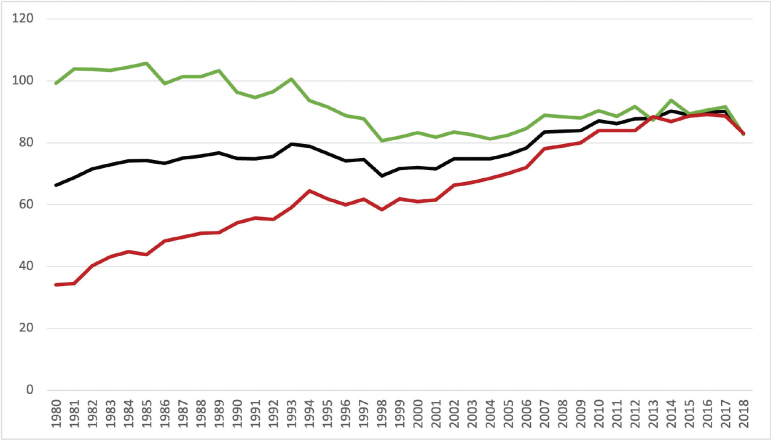
Incidence of lung cancer per 100,000 inhabitants (Black line: All incidence; Green line: Male incidence; Red line: Female incidence).

Figure 2 depicts the crude incidence of lung cancer per 100,000 inhabitants as well as separately for males and females. Analyzing the gender-specific differences, the incidence rate among males declined from a peak of 106 per 100,000 in 1985 to a consistent range between 81 and 92 per 100,000 inhabitants from 1998 to 2018. Among female lung cancer patients, the incidence rate exhibited an ongoing upward trajectory, rising from a low rate of 34 per 100,000 inhabitants in 1980 to a peak of 89 per 100,000 inhabitants in 2015.

**Figure 3:**
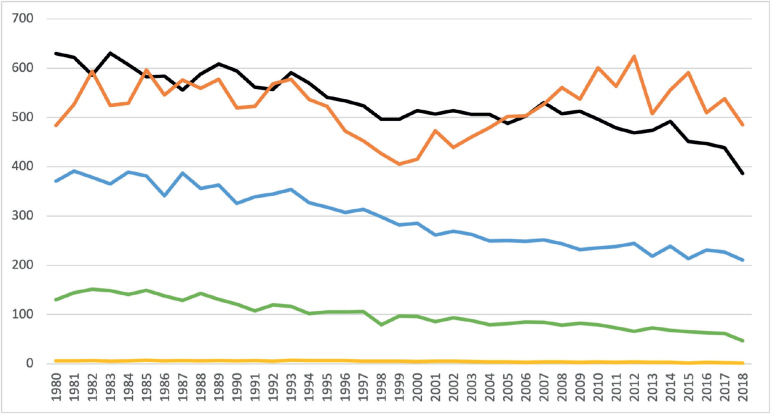
Incidence of lung cancer per 100,000 inhabitants among males (Yellow line: males <50 years; Green line: males 50–59 years; Blue line: males 60–69 years; Black line: males 70–79 years; Orange line: males >80 years).

Patients were categorized into age ranges: below 50 years, 50–59 years, 60–69 years, 70–79 years, and 80 years and above. Figure 3 showcases the evolution of lung cancer incidence within male age groups. Incidence remained stable for males under 50 years, hovering around 2–6 per 100,000 inhabitants. For males aged 50–59, 60–69, and 70–79 years, noticeable declines in incidence were observed, plummeting from approximately 150–50 per 100,000 inhabitants (50–59 years), 390–210 per 100,000 inhabitants (60–69 years), and 630–390 per 100,000 inhabitants (70–79 years). Notably, lung cancer incidence among males above 80 years experienced an initial decline from 600 to 400 per 100,000 inhabitants in the 1980s, followed by an increase that peaked in 2012 at 624 per 100,000 inhabitants.

**Figure 4:**
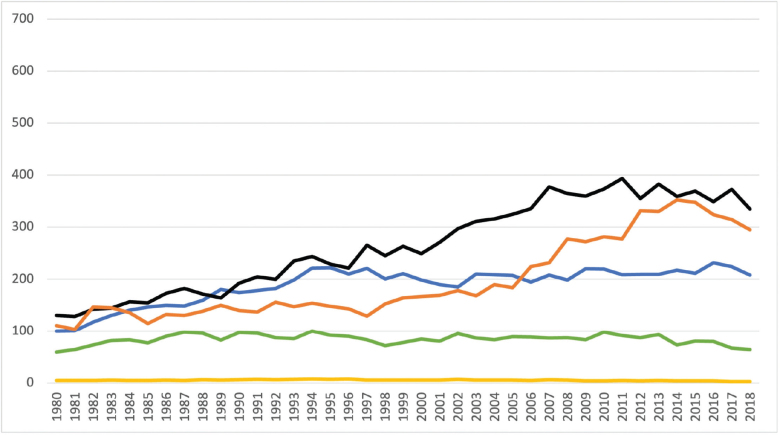
Incidence of lung cancer per 100,000 inhabitants among females (Yellow line: females <50 years; Green line: females 50–59 years; Blue line: females 60–69 years; Black line: females 70–79 years; Orange line: females >80 years).

The age-specific incidence of lung cancer of females is shown in Figure 4. For females, the lung cancer incidence remained steady within age groups below 50 years (3–7 per 100,000 inhabitants) and 50–59 years (60–100 per 100,000 inhabitants). In the 60–69 years age group, a notable increase was observed, climbing from 100 to 230 per 100,000 inhabitants over the study period. Age groups 70–79 years and 80 years and above exhibited distinct increases in incidence over time, rising from 130 to 400 per 100,000 inhabitants (age group 70–79) and from 100 to 350 per 100,000 inhabitants (age group 80 and above).

## Discussion

Our study presents an overview of sex- and age-specific incidence rates of lung cancer and smoking prevalence trends in Denmark over several decades. Notably, although a peak in the crude incidence rate was observed in 2014–2017, no significant difference in incidence rate was noted before and after the peak, and there is no clear indication that lung cancer incidence is currently declining. Furthermore, there has been a significant shift in the sex distribution of patients, transitioning from a male majority to an equal male-female ratio in recent years. The analysis also highlights changes in age distribution, indicating a decline in patients aged 50–59 and an increase in those aged above 80. Regarding smoking prevalence, the male population has exhibited a consistent decline from 1950 to 2000. In contrast, during the same period, female smoking prevalence initially increased but has shown a decline from the late 1980s to 2020.

There was no significant difference observed in the crude incidence of lung cancer before and after the peak in the years 2014–2017. Currently, it remains uncertain whether a downward trend in lung cancer incidence has commenced. Considering the history of smoking prevalence and the estimated 40–50-year time lag between smoking initiation and the diagnosis of lung cancer, a decrease in the incidence of lung cancer, especially among females, is unlikely to occur for a couple of decades. This is attributed to the fact that smoking prevalence was relatively stable in the 1980s, and the anticipated increase in life expectancy supports this perspective [17, 18]. Given the high incidence of lung cancer among females, consideration should be given to implementing specific smoking cessation programs targeting women [19]. Cigarette smoking prevalence has only seen a small decline over the last decade, and ensuring a continued decline in lung cancer incidence necessitates the initiation of legislation aimed at discouraging young individuals from starting smoking as suggested in certain countries adopting the concept of the tobacco endgame [20].

The incidence rate of lung cancer in women in Denmark is now equal to that of men, even when accounting for differences in life expectancy, as demonstrated by the similar trends in age-standardized incidence rate [21]. Although Danish women have a high prevalence of smoking at a global level [22], the prevalence of smoking has never exceeded that of men. A possible explanation for this observation is that women are more susceptible to developing lung cancer than men, also within the Caucasian population. This possibility is supported by the fact that the majority of heavy smokers between 1970 and 2002 were male [16] and could be attributed to lower lung size among women and sex-related differences in airway behavior [23]. Alternatively, women might be more likely to identify themselves as never-smokers at the hospital, even if they are current or previous smokers, due to the greater stigma attached to smoking for women than for men in certain cultures and social contexts. This is substantiated by a prior discovery that approximately 20% of pregnant women attending smoking cessation programs were registered as non-smokers in the National Birth Registry [24].

Over the course of four decades, there has been a notable decline in the proportion of diagnosed lung cancer patients aged under 50, dropping from 5.7% during the period 1980–1993 to 2.6% in the period 2008–2018. Moreover, crude incidence rates within this age group, for both males and females, have exhibited a similar decreasing trend, plummeting from 6 cases per 100,000 individuals in 1980 to 2–3 cases per 100,000 individuals in 2018. Previous characteristics of young lung cancer patients have indicated that this subgroup shared smoking rates with their older counterparts. Additionally, females were more prevalent among young lung cancer patients, and adenocarcinoma was the predominant histologic subtype [25, 26]. Notably, young lung cancer patients often harbor a higher frequency of driver mutations, including epidermal growth factor receptor (*EGFR*) and anaplastic lymphoma kinase (*ALK*) translocations [27]. The decrease in the number of young lung cancer patients is likely attributable to the concurrent reduction in cigarette smoking exposure observed in the younger population [16].

### Perspectives

Considering historical smoking trends and the substantial time lag between smoking initiation and lung cancer diagnosis, a decline in female lung cancer incidence is unlikely for several decades. To address this, targeted smoking cessation programs for women are crucial. Legislation discouraging smoking initiation among youth is essential to sustain lung cancer incidence reduction. With Western countries experiencing aging populations and declining smoking rates, the median age of lung cancer patients is expected to rise [17, 18], emphasizing the need for tailored diagnostic [28] and treatment strategies. Future research should prioritize early detection, minimally invasive diagnostics, and personalized treatments to accommodate the needs of a growing frail population [29].

### Strengths and limitations

One advantage of the present cohort study is its comprehensive coverage of nationwide lung cancer incidence in Denmark, using the well-established validity of the Danish Cancer Registry [30]. However, the inherent limitations of epidemiological study design mean that detailed information about individual lung cancer patients is not accessible. Consequently, the underlying reasons for the elevated incidence of lung cancer in females, despite their lower cigarette smoking prevalence compared to males, remain speculative. The study is expected to demonstrate high external validity among European individuals with similar ethnic demographics.

## Conclusions

In summary, our study offers a comprehensive analysis of the sex and age distribution of lung cancer over a four-decade span at the national level. A decline in lung cancer incidence within the decade is uncertain. Notably, female lung cancer patients have surpassed males in incidence, despite lower rates of cigarette smoking. The age distribution trends are evident, pointing to an anticipated rise in lung cancer cases among the elderly population in the future. This scenario underscores the need for increasingly personalized diagnostic methods and treatment options to address the unique characteristics of this aging and potentially frail subgroup.

## Data Availability

Anonymized data will be provided by the corresponding author at reasonable request.

## References

[1] Mattiuzzi C, Lippi G. Current cancer epidemiology. J Epidemiol Glob Health. 2019;9(4):217. 10.2991/jegh.k.191008.001 31854162 PMC7310786

[2] Parkin DM, Pisani P, Ferlay J. Estimates of the worldwide incidence of eighteen major cancers in 1985. Int J Cancer. 1993;54(4):594–606. 10.1002/ijc.2910540413 8514451

[3] Giovino GA, Henningfield JE, Tomar SL, et al. Epidemiology of tobacco use and dependence. Epidemiol Rev. 1995;17(1):48–65. 10.1093/oxfordjournals.epirev.a036185 8521946

[4] Samarasekera U. WHO’s ninth report on the global tobacco epidemic. Lancet Oncol. 2023;24(9):957. 10.1016/S1470-2045(22)00341-2 37544312

[5] Funatogawa I, Funatogawa T, Yano E. Impacts of early smoking initiation: long-term trends of lung cancer mortality and smoking initiation from repeated cross-sectional surveys in Great Britain. BMJ Open. 2012;2(5):e001676. 10.1136/bmjopen-2012-001676 PMC3488725 23048061

[6] National Center for Chronic Disease Prevention and Health Promotion (US) Office on Smoking and Health. *The health consequences of smoking – 50 years of progress: a report of the surgeon general* [Internet]. Atlanta, GA: Centers for Disease Control and Prevention (US); 2014. (Reports of the Surgeon General). Available from: http://www.ncbi.nlm.nih.gov/books/NBK179276/24455788

[7] Chaitanya Thandra K, Barsouk A, Saginala K, et al. Epidemiology of lung cancer. Współczesna Onkol. 2021;25(1):45–52. 10.5114/wo.2021.103829 PMC8063897 33911981

[8] Wang XR, Chiu YL, Qiu H, et al. The roles of smoking and cooking emissions in lung cancer risk among Chinese women in Hong Kong. Ann Oncol. 2009;20(4):746–51. 10.1093/annonc/mdn699 19150939

[9] Giaquinto AN, Miller KD, Tossas KY, et al. Cancer statistics for African American/Black People 2022. CA Cancer J Clin. 2022;72(3):202–29. 10.3322/caac.21718 35143040

[10] Haiman CA, Stram DO, Wilkens LR, et al. Ethnic and racial differences in the smoking-related risk of lung cancer. N Engl J Med. 2006;354(4):333–42. 10.1056/NEJMoa033250 16436765

[11] In KH, Kwon YS, Oh IJ, et al. Lung cancer patients who are asymptomatic at diagnosis show favorable prognosis: a Korean Lung Cancer Registry Study. Lung Cancer. 2009;64(2):232–7. 10.1016/j.lungcan.2008.08.005 18809225

[12] Skuladottir H, Olsen JH, Hirsch FR. Incidence of lung cancer in Denmark: historical and actual status. Lung Cancer. 2000;27(2): 107–18. 10.1016/S0169-5002(99)00104-X 10688493

[13] Lynge E, Sandegaard JL, Rebolj M. The Danish National Patient Register. Scand J Public Health. 2011;39(7_Suppl.):30–3. 10.1177/1403494811401482 21775347

[14] Schmidt M, Schmidt SAJ, Sandegaard JL, et al. The Danish National Patient Registry: a review of content, data quality, and research potential. Clin Epidemiol. 2015;7:449–90. 10.2147/CLEP.S91125 26604824 PMC4655913

[15] World Health Organization. (2004). ICD-10 : international statistical classification of diseases and related health problems : tenth revision, 2nd ed. World Health Organization. https://iris.who.int/handle/10665/42980 3376487

[16] Danish Health Authority. *Tobaksrygning og rygestop: Konsekvenser for sundheden* [Internet]. [Cited date: December 14th, 2023] Available from: https://www.sst.dk/~/media/39CD08DAD84C4727AA7408A643C3A1E0.ashx

[17] Statistics Denmark [Internet]. [Cited date: December 14th, 2023] Available from: https://www.statistikbanken.dk/10022

[18] Vespa J, Medina L, Armstong D. *Demographic turning points for the United States: population projections for 2020 to 2060* [Internet]. [Cited date: December 14th, 2023] Available from: https://www.census.gov/content/dam/Census/library/publications/2020/demo/p25-1144.pdf

[19] Manns A, Torregrossa H, Mahdjoub S, et al. Do determinants of smoking cessation and relapse differ between men and women? Data from a French National Study. Subst Use Misuse. 2024;59(2):167–76. 10.1080/10826084.2023.2267106 37813814

[20] Kang H, Cheon E, Kim HK, et al. Vision for tobacco endgame in Korea: suggestions for countries with endgame aspirations. Tob Control. 2023:tc-2022-057691. 10.1136/tc-2022-057691 37147128

[21] Engholm G, Ferlay J, Christensen N, et al. NORDCAN – a Nordic tool for cancer information, planning, quality control and research. Acta Oncol. 2010;49(5):725–36. 10.3109/02841861003782017 20491528

[22] Jafari A, Rajabi A, Gholian-Aval M, et al. National, regional, and global prevalence of cigarette smoking among women/females in the general population: a systematic review and meta-analysis. Environ Health Prev Med. 2021;26(1):5. 10.1186/s12199-020-00924-y 33419408 PMC7796590

[23] Becklake MR, Kauffmann F. Gender differences in airway behaviour over the human life span. Thorax. 1999;54(12):1119–38. 10.1136/thx.54.12.1119 10567633 PMC1763756

[24] Rasmussen M, Tønnesen H. Pregnant smokers: potential for improvement of intervention. Clin Health Promot Res Best Pract. 2015;5(3):67–73. 10.29102/clinhp.150010

[25] Tominaga K, Mori K, Yokoi K, et al. Lung cancer in patients under 50 years old. Jpn J Cancer Res. 1999;90(5):490–5. 10.1111/j.1349-7006.1999.tb00774.x 10391087 PMC5926099

[26] Veness M, Delaney G, Berry M. Lung cancer in patients aged 50 years and younger: clinical characteristics, treatment details and outcome. Aust Radiol. 1999;43(3):328–33. 10.1046/j.1440-1673.1999.433666.x 10901928

[27] Suidan AM, Roisman L, Belilovski Rozenblum A, et al. Lung cancer in young patients: higher rate of driver mutations and brain involvement, but better survival. J Glob Oncol. 2019;5:1–8. 10.1200/JGO.18.00216 PMC6550091 31067141

[28] O’Donovan A, Leech M. Personalised treatment for older adults with cancer: the role of frailty assessment. Tech Innov Patient Support Radiat Oncol. 2020;16:30–8. 10.1016/j.tipsro.2020.09.001 33102819 PMC7568178

[29] Leduc C, Antoni D, Charloux A, et al. Comorbidities in the management of patients with lung cancer. Eur Respir J. 2017;49(3):1601721. 10.1183/13993003.01721-2016 28356370

[30] Gjerstorff ML. The Danish Cancer Registry. Scand J Public Health. 2011;39(7_Suppl.):42–5. 10.1177/1403494810393562 21775350

